# ICTV Virus Taxonomy Profile: *Ophioviridae*

**DOI:** 10.1099/jgv.0.000836

**Published:** 2017-06-21

**Authors:** María Laura García, Elena Dal Bó, John V. da Graça, Selma Gago-Zachert, John Hammond, Pedro Moreno, Tomohide Natsuaki, Vicente Pallás, Jose A. Navarro, Carina A. Reyes, Gabriel Robles Luna, Takahide Sasaya, Ioannis E. Tzanetakis, Anna María Vaira, Martin Verbeek, ICTV Report Consortium

**Affiliations:** ^1^​ Instituto de Biotecnología y Biología Molecular, Universidad de La Plata, La Plata, Argentina; ^2^​ Facultad de Ciencias Agrarias y Forestales, Universidad de La Plata, La Plata, Argentina; ^3^​ Texas A and M University-Kingsville, Citrus Center, Weslaco, USA; ^4^​ Department of Molecular Signal Processing, Leibniz Institute of Plant Biochemistry, Halle (Saale), Germany; ^5^​ U.S. Department of Agriculture, Agricultural Research Service, Beltsville, Maryland, USA; ^6^​ Centro de Protección Vegetal y Biotecnología, Instituto Valenciano de Investigaciones, Agrarias, Moncada, Valencia, Spain; ^7^​ Faculty of Agriculture, Utsunomiya University, Utsunomiya, Japan; ^8^​ Instituto de Biología Molecular y Celular de plantas (IBMCP), Universidad Politécnica de Valencia-Consejo Superior de Investigaciones Científicas, Valencia, Spain; ^9^​ Department of Planning and Coordination, National Agriculture and Food Research Organization, Tsukuba, Japan; ^10^​ Department of Plant Pathology, Division of Agriculture, University of Arkansas, USA; ^11^​ Institute for Sustainable Plant Protection (IPSP) – CNR, Torino, Italy; ^12^​ Wageningen Plant Research, Wageningen University and Research, Wageningen, The Netherlands

**Keywords:** *Ophioviridae*, ICTV, taxonomy, citrus psorosis virus, Mirafiori lettuce big-vein virus, lettuce ring necrosis virus, blueberry mosaic associated virus

## Abstract

The *Ophioviridae* is a family of filamentous plant viruses, with single-stranded negative, and possibly ambisense, RNA genomes of 11.3–12.5 kb divided into 3–4 segments, each encapsidated separately. Virions are naked filamentous nucleocapsids, forming kinked circles of at least two different contour lengths. The sole genus, *Ophiovirus*, includes seven species. Four ophioviruses are soil-transmitted and their natural hosts include trees, shrubs, vegetables and bulbous or corm-forming ornamentals, both monocots and dicots. This is a summary of the International Committee on Taxonomy of Viruses (ICTV) Report on the taxonomy of the *Ophioviridae,* which is available at http://www.ictv.global/report/ophioviridae.

## Abbreviations

CP, coat protein; RdRp, RNA-dependent RNA polymerase.

## Virion

**Fig. 1.**
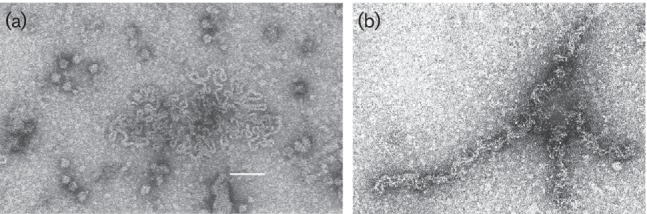
Negative contrast electron micrographs (uranyl acetate) of virus particles. Bar, 100 nm. (a) Open form of the large particle (citrus psorosis virus), and (b) collapsed form (freesia sneak virus) (courtesy of R. G. Milne).

Particles are non-enveloped, naked filamentous nucleocapsids forming either circles (open form) or pseudo-linear duplex (collapsed form) (Table 1, Fig. 1).

**Table 1.** Characteristics of the family *Ophioviridae*

Typical member:	citrus psorosis virus P-121 (RNA1 AY654892; RNA2: AY654893; RNA3: AY654894), species *Citrus psorosis virus*, genus *Ophiovirus*
Virion	Non-enveloped, nucleocapsids 3 nm in diameter, and 700 nm or 2000 nm long. Pseudo-linear duplex structures are 9–10 nm in diameter
Genome	11.3–12.5 kb of negative-sense, segmented RNA (3 or 4 segments)
Replication	Not characterized
Translation	From mRNAs, which are complementary to the vRNAs
Host range	Citrus, blueberry, pittosporum, lettuce, sowthistle, tulip, ranunculus, anemone, lachenalia and freesia
Taxonomy	One genus, *Ophiovirus,* including seven species

## Genome

**Fig. 2.**
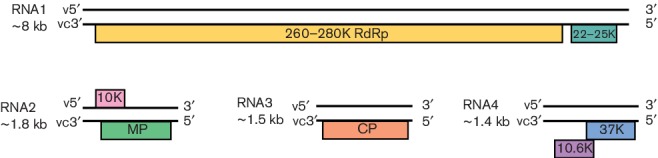
Genome organization of the genus *Ophiovirus*. Mirafiori lettuce big-vein virus is shown (modified from [2]). Boxes represent ORFs. The length of the RNA segments and the predicted sizes of the ORF products are indicated. RNA4 is not reported for all ophioviruses. v, viral RNA; vc, viral complementary RNA; RdRp, RNA polymerase; MP, movement protein; CP, coat protein.

The genome of ophioviruses consists of three or four individually encapsidated RNA segments (Fig. 2). Members of the species *Citrus psorosis virus* [1], *R* *anunculus white mottle virus*, *F* *reesia sneak virus* and *B* *lueberry mosaic associated virus* have three RNAs (named RNA1, RNA2 and RNA3) whereas members of the species *Mirafiori lettuce big-vein virus* and *Lettuce ring necrosis virus* have a fourth RNA (RNA4) [2]. The genome organization is unknown for members of the species *Tulip mild mottle mosaic virus*.

Virions encapsidate both the minus- and positive-sense RNAs, but a larger amount of RNA of negative polarity is detected. As virions appear circularized, the presence of panhandle structures has been suggested. RNA1 contains two ORFs in the viral complementary strand (vcRNA) encoding the 22–25K protein and the RdRp. The 24K protein of citrus psorosis virus is involved in the misprocessing of miRNA and RNA silencing suppressor activity. vcRNA2 encodes the cell-to-cell movement protein (MP), which also has RNA silencing suppressor activity. Mirafiori lettuce big-vein virus has a second putative ORF in vRNA2 that encodes a protein of unknown function. The coat protein (CP) is encoded by vcRNA3. A fourth genomic RNA has been reported for Mirafiori lettuce big-vein virus and lettuce ring necrosis virus, encoding putative proteins of unknown function.

## Taxonomy

*Ophiovirus* is the only genus in the family, including seven species. CP amino acid sequence alignments show 31–52 % identity between isolates of different species, and 92–100 % identity within a species. The CPs of Mirafiori lettuce big-vein virus and tulip mild mottle mosaic virus (partial CDS) share about 80 % amino acid sequence identity, thus warranting an ophiovirus species demarcation criterion of <85 % CP amino acid sequence identity. The RdRp contains the SDD sequence in motif C, a signature for segmented negative-stranded RNA viruses. Phylogenetic reconstructions using sequences of the conserved core module from RdRps of ophioviruses and representative negative-stranded RNA viruses reinforce their separation as a monophyletic group. Citrus psorosis virus is present worldwide, transmitted by vegetative propagation of the host, and natural dispersion has also been observed. No vector is known for ranunculus white mottle virus and blueberry mosaic associated virus. *Olpidium virulentus* and *Olpidium brassicae* fungi transmit Mirafiori lettuce big-vein virus, tulip mild mottle mosaic virus and lettuce ring necrosis virus; freesia sneak virus is presumably transmitted by a member of the genus *Olpidium*. Ranunculus white mottle virus has been reported in Italy, France and Germany [3], and tulip mild mottle mosaic virus in Japan [4]. Mirafiori lettuce big-vein virus, the causal agent of big-vein disease in lettuce, probably occurs worldwide. Lettuce ring necrosis virus is closely associated with lettuce ring necrosis disease in The Netherlands, Belgium and France [5]. Freesia sneak virus [6] has been reported in Europe, South Africa, North America, South Korea and New Zealand. Blueberry mosaic associated virus is associated with blueberry mosaic disease found in North and South America, Europe, New Zealand and Japan [7].

## Resource

Full ICTV Online (10th) Report: www.ictv.global/report/ophioviridae.
