# Corrigendum: ICTV Virus Taxonomy Profile: *Ophioviridae*

**DOI:** 10.1099/jgv.0.001093

**Published:** 2018-07-02

**Authors:** María Laura García, Elena Dal Bó, John V. da Graça, Selma Gago-Zachert, John Hammond, Pedro Moreno, Tomohide Natsuaki, Vicente Pallás, Jose A. Navarro, Carina A. Reyes, Gabriel Robles Luna, Takahide Sasaya, Ioannis E. Tzanetakis, Anna María Vaira, Martin Verbeek, ICTV Report Consortium

**Affiliations:** ^1^​ Instituto de Biotecnología y Biología Molecular, Universidad de La Plata, La Plata, Argentina; ^2^​ Facultad de Ciencias Agrarias y Forestales, Universidad de La Plata, La Plata, Argentina; ^3^​ Texas A and M University-Kingsville, Citrus Center, Weslaco, USA; ^4^​ Department of Molecular Signal Processing, Leibniz Institute of Plant Biochemistry, Halle (Saale), Germany; ^5^​ U.S. Department of Agriculture, Agricultural Research Service, Beltsville, Maryland, USA; ^6^​ Centro de Protección Vegetal y Biotecnología, Instituto Valenciano de Investigaciones, Agrarias, Moncada, Valencia, Spain; ^7^​ Faculty of Agriculture, Utsunomiya University, Utsunomiya, Utsunomiya, Japan; ^8^​ Instituto de Biología Molecular y Celular de plantas (IBMCP), Universidad Politécnica de Valencia-Consejo Superior de Investigaciones Científicas, Valencia, Spain; ^9^​ Department of Planning and Coordination, National Agriculture and Food Research Organization, Tsukuba, Japan; ^10^​ Department of Plant Pathology, Division of Agriculture, University of Arkansas, USA; ^11^​ Institute for Sustainable Plant Protection (IPSP) – CNR, Torino, Italy; ^12^​ Wageningen Plant Research, Wageningen University and Research, Wageningen, The Netherlands

**Keywords:** citrus psorosis virus, lettuce ring necrosis virus, Mirafiori lettuce big-vein virus, ICTV, blueberry mosaic associated virus, taxonomy, *Ophioviridae*, *Aspiviridae*

The International Committee on Taxonomy of Viruses has ratified a taxonomic proposal to change the name of the family *Ophioviridae* to *Aspiviridae*, and to change the names of species in the family to *Blueberry mosaic associated ophiovirus*, *Citrus psorosis ophiovirus, Freesia sneak ophiovirus, Lettuce ring necrosis ophiovirus, Mirafiori lettuce big-vein ophiovirus, Ranunculus white mottle ophiovirus* and *Tulip mild mottle mosaic ophiovirus*. These species are all members of the genus *Ophiovirus*, the genus name being unchanged.

The ICTV Online (10th) Report on the family *Aspiviridae* can be found at www.ictv.global/report/aspiviridae.

